# The Silencing of Carotenoid β-Hydroxylases by RNA Interference in Different Maize Genetic Backgrounds Increases the β-Carotene Content of the Endosperm

**DOI:** 10.3390/ijms18122515

**Published:** 2017-11-24

**Authors:** Judit Berman, Uxue Zorrilla-López, Gerhard Sandmann, Teresa Capell, Paul Christou, Changfu Zhu

**Affiliations:** 1 Department of Plant Production and Forestry Science, University of Lleida-Agrotecnio Center, Av. Alcalde Rovira Roure, 191, 25198 Lleida, Spain; jberman@hbj.udl.cat (J.B.); uxue87@gmail.com (U.Z.-L.); teresa.capell@pvcf.udl.cat (T.C.); christou@pvcf.udl.cat (P.C.); 2 Biosynthesis Group, Molecular Biosciences, Johann Wolfgang Goethe Universität, 60054 Frankfurt, Germany; sandmann@bio.uni-frankfurt.de; 3 ICREA, Catalan Institute for Research and Advanced Studies, Passeig Lluís Companys 23, 08010 Barcelona, Spain; 4 School of Life Sciences, Changchun Normal University, Changchun 130032, China

**Keywords:** maize (*Zea mays* L.), β-carotene, carotenoid β-hydroxylase, RNAi, hybrid

## Abstract

Maize (*Zea mays* L.) is a staple food in many parts of Africa, but the endosperm generally contains low levels of the pro-vitamin A carotenoid β-carotene, leading to vitamin A deficiency disease in populations relying on cereal-based diets. However, maize endosperm does accumulate high levels of other carotenoids, including zeaxanthin, which is derived from β-carotene via two hydroxylation reactions. Blocking these reactions could therefore improve the endosperm β-carotene content. Accordingly, we used RNA interference (RNAi) to silence the endogenous *ZmBCH1* and *ZmBCH2* genes, which encode two non-heme di-iron carotenoid β-hydroxylases. The genes were silenced in a range of maize genetic backgrounds by introgressing the RNAi cassette, allowing us to determine the impact of *ZmBCH1*/*ZmBCH2* silencing in diverse hybrids. The β-carotene content of the endosperm increased substantially in all hybrids in which *ZmBCH2* was silenced, regardless of whether or not *ZmBCH1* was silenced simultaneously. However, the β-carotene content did not change significantly in C17 hybrids (M7 × C17 and M13 × C17) compared to C17 alone, because *ZmBCH2* is already expressed at negligible levels in the C17 parent. Our data indicate that *ZmBCH2* is primarily responsible for the conversion of β-carotene to zeaxanthin in maize endosperm.

## 1. Introduction

Humans are unable to synthesize vitamin A de novo and must obtain this essential nutrient in their diet, either directly as retinol from animal sources or as pro-vitamin A (PVA) carotenoids from plants. The major PVA carotenoid is β-carotene, which can be converted into two molecules of retinol. The other PVA carotenoids are α-carotene, γ-carotene, and β-cryptoxanthin, and these yield one molecule of retinol each [1]. Vitamin A in its reduced form (retinal) is necessary for rhodopsin biosynthesis, making this nutrient essential for normal vision, and it also helps to maintain healthy epithelial and immune system cells. The acidic form (retinoic acid) is a key developmental morphogen. Therefore, vitamin A deficiency (VAD) causes visual disorders in the form of night blindness and (in more serious cases) xerophthalmia, as well as epithelial and immune system defects that leave individuals, especially children, highly susceptible to infections [1,2,3]. The polished grain (endosperm) of cereal crops such as rice (*Oryza sativa*), maize (*Zea mays*), and wheat (*Triticum aestivum*) are staples in many parts of the world, but they contain very low levels of PVA carotenoids, hence populations that subsist on cereal-rich diets are prone to VAD [4,5]. Increasing the levels of PVA carotenoids in cereals is an effective way to combat this challenge [1,2,4,6,7].

**Figure 1:**
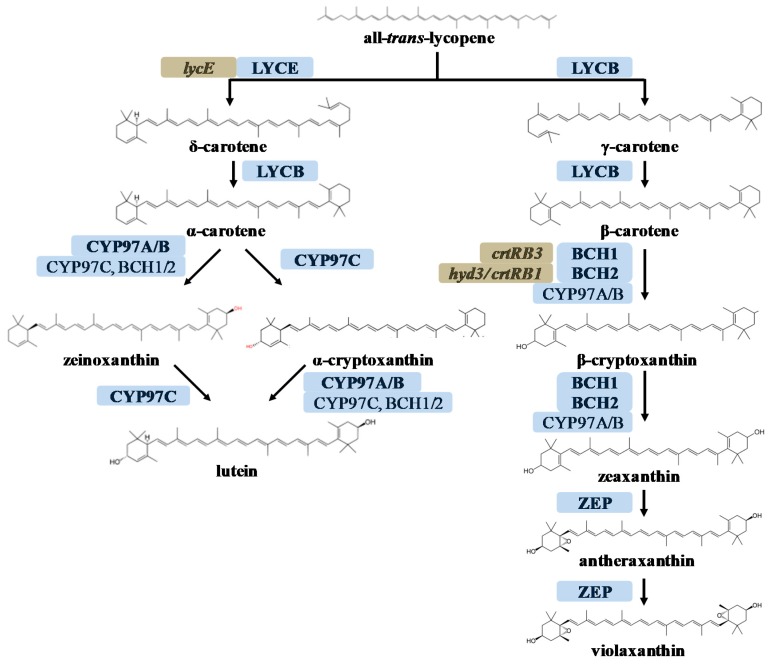
Xanthophyll biosynthesis pathway in maize endosperm. Enzymes responsible for the major activity at each step are shown in bold (blue). Identified maize locus names corresponding to each enzyme are shown in brown. Abbreviations: LYCB, lycopene β-cyclase; LYCE, lycopene ε-cyclase; CYP97C, heme-containing cytochrome P450 carotenoid ε-ring hydroxylase; CYP97A/B, heme-containing cytochrome P450 carotenoid β-ring hydroxylase; BCH1/2, Non-heme di-iron β-carotenoid hydroxylases; ZEP, zeaxanthin epoxidase.

PVA carotenoids are synthesized via the methylerythritol 4-phosphate (MEP) pathway, which is also known as the non-mevalonic acid (MVA) pathway [6,7,8]. The first committed step in carotenoid biosynthesis is the condensation of two molecules of geranylgeranyl diphosphate (GGPP) by phytoene synthase (PSY) to produce phytoene, which is then converted into all-*trans*-lycopene via four desaturation and isomerization steps. Lycopene is the branching point of this pathway (Figure 1). In one branch, lycopene is cyclized to form the PVA carotenoids γ-carotene and β-carotene. The latter is oxidized to form another PVA carotenoid (β-cryptoxanthin) and subsequently the non-PVA carotenoid zeaxanthin, antheraxanthin, and violaxanthin. In the other branch, lycopene is cyclized to produce the PVA carotenoid α-carotene, which is oxidized to form the non-PVA carotenoids α-cryptoxanthin and zeinoxanthin, and finally lutein. The oxidation reactions that deplete the pool of PVA carotenoids are catalyzed by enzymes known as carotenoid β-hydroxylases.

Two pairs of duplicated carotenoid hydroxylases have been identified in *Arabidopsis thaliana* [9,10], tomato [11,12,13], rice [14,15], and maize [16,17,18,19,20]. These can be classified as non-heme di-iron hydroxylases (BCH type) and heme-containing cytochrome P450 hydroxylases (CYP type). The BCH type enzymes BCH1 and BCH2 are primarily responsible for the hydroxylation of β-carotene and other β,β-carotenoids, although CYP-type enzymes can oxidize the β-rings of β-carotene to some extent in the *A. thaliana bch1 bch2* double mutant [9], and other CYP-type enzymes have also been shown to hydroxylate α-carotene and/or β-carotene in plants and *Escherichia coli* [10,14]. For example, the tomato (*Solanum lycopersicum*) BCH-type enzymes CRTR-B1 and CRTR-B2, as well as CYP97A29, can hydroxylate the β-ring of α-carotene [11,12,13].

Two maize *BCH* genes (*ZmBCH1* and *ZmBCH2*) (*ZmBCH1,* also termed *ZmcrtRB3,* or *ZmHYD4; ZmBCH2* also termed *ZmcrtRB1,* or *ZmHYD3*) have been cloned and functionally characterized [16,17,18,19]. Candidate-gene association analysis identified 18 polymorphic sites in *ZmBCH1* significantly associated with one or more carotenoid-related traits in 126 diverse yellow maize inbred lines, indicating that *ZmBCH1* plays a role in hydroxylating the β-rings of α-carotene and β-carotene, but predominantly α-carotene [19]. In contrast, quantitative trait locus (QTL) mapping, genome-wide association studies (GWAS), and functional analysis revealed that *ZmBCH2* strongly influences the conversion of β-carotene to zeaxanthin, with hypomorphic *ZmBCH2* alleles correlating with higher β-carotene levels in the endosperm [16,18,21,22]. *ZmBCH2* is also the only carotenoid hydroxylase gene expressed at high enough levels in maize endosperm to be detected by mRNA blot, and it is preferentially transcribed in the endosperm [17].

In potato (*Solanuum tuberosum*), silencing the *BCH1* gene by RNA interference (RNAi) increased β-carotene levels from trace amounts in wild-type tubers to 3.31 µg/g fresh weight in the transgenic plants, with a concomitant loss of zeaxanthin and an increase in lutein [23]. The simultaneous silencing of both potato *BCH* genes increased β-carotene levels by up to 38-fold (with a concomitant loss of zeaxanthin) and the total carotenoid level increased by up to 4.5-fold [24]. Similarly, silencing *BCH* expression in sweet orange plants (*Citrus sinensis*) produced oranges with a deep yellow color and up to 36-fold more β-carotene [25]. In wheat, blocking the expression of an endogenous *BCH* gene promoted the conversion of β-carotene into xanthophylls, but nevertheless increased the β-carotene levels in the endosperm by up to 10-fold [26].

The ability to increase β-carotene levels in other crops by silencing one or more *BCH* genes suggests that a similar strategy could be deployed in maize to address VAD in developing country populations. However, given the diverse activities of carotenoid β-hydroxylases in different crops, a functional analysis of the corresponding genes is necessary during the development of biotechnology programs to increase PVA levels. This is particularly important in maize due to the low ratio of PVA to non-PVA carotenoids in the endosperm and the diverse carotenoid profiles of different maize varieties [27]. In order to evaluate the impact of silencing both of the *BCH*-type enzymes in different genetic backgrounds, we therefore introgressed *ZmBCH1/2* RNAi silencing cassettes into different lines and investigated the endosperm carotenoid content in the resulting hybrids.

## 2. Results

### 2.1. RNAi-Mediated Silencing of ZmBCH1 and ZmBCH2 in M37W White Maize

**Figure 2:**
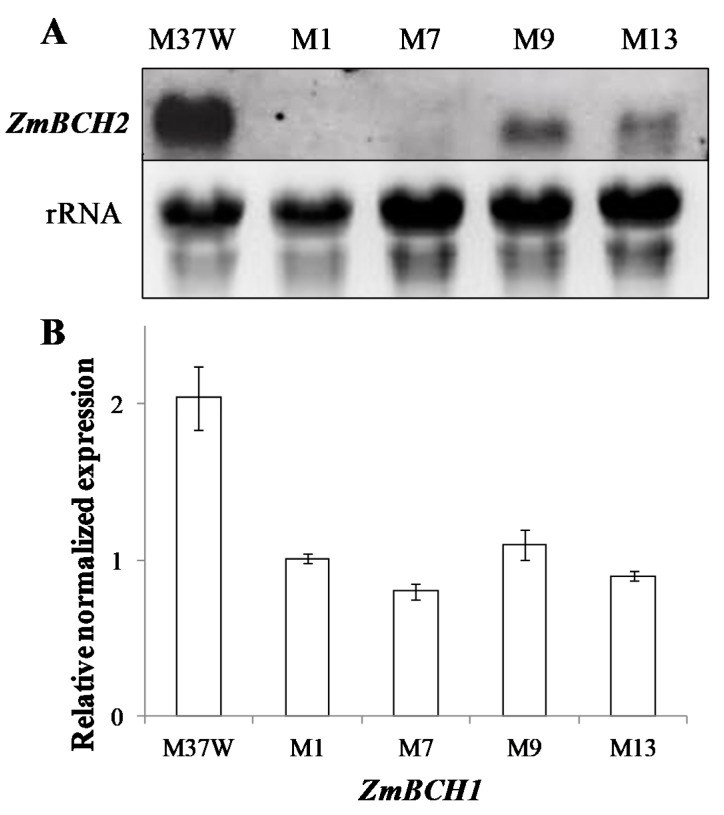
*BCH* gene expression in the endosperm of wild-type (M37W) maize and independent transgenic lines M1, M7, M9, and M13 at 30 DAP. (**A**) An mRNA blot (25 µg of total RNA per lane) to monitor *ZmBCH2* expression; (**B**) Endogenous *ZmBCH1* mRNA levels detected by qPCR presented as means of three replicates ± SE (standard error).

The *ZmBCH1* and *ZmBCH2* DNA fragments used for RNAi construct show 96.4% identity, so the pHorP-RNAi-ZmBCH2 silencing construct was expected to downregulate not only the primary target *ZmBCH2* but also the closely related paralog *ZmBCH1* in M37W maize. This was confirmed by mRNA analysis. T3 endosperm from homozygous T2 plants was analyzed using selective probes for each transcript, revealing negligible levels of endogenous *ZmBCH2* mRNA in two transgenic lines (M1 and M7) and low levels in two additional lines (M9 and M13) (Figure 2A). The *ZmBCH1* transcript could not be detected in any of these lines by mRNA blot analysis so quantitative RT-PCR was used instead. This revealed that *ZmBCH1* was downregulated in all transgenic lines by at least two-fold, compared to M37W wild-type plants (Figure 2B).

### 2.2. Carotenoid Profiles in Hybrids Derived from BCH-Silenced Parents

Transgenic lines M7 and M13 were chosen to represent lines with near-complete *ZmBCH2* silencing and partial *ZmBCH2* silencing, respectively. Each transgenic line was crossed with a range of maize inbred lines to generate hybrids in different genetic backgrounds. The wild-type inbred parents were selected based on their diverse carotenoid profiles (Table S2). Inbred lines B73 and C17 accumulated lutein as the predominant carotenoid in the endosperm, whereas lines NC356, O1-3, O2-9, and PSY1 accumulated zeaxanthin instead. The purpose of these experiments was to investigate the impact of partial and near-complete *ZmBCH* gene silencing in diverse genetic backgrounds representing different expression levels of genes in the endogenous carotenoid biosynthesis pathway.

**Figure 3:**
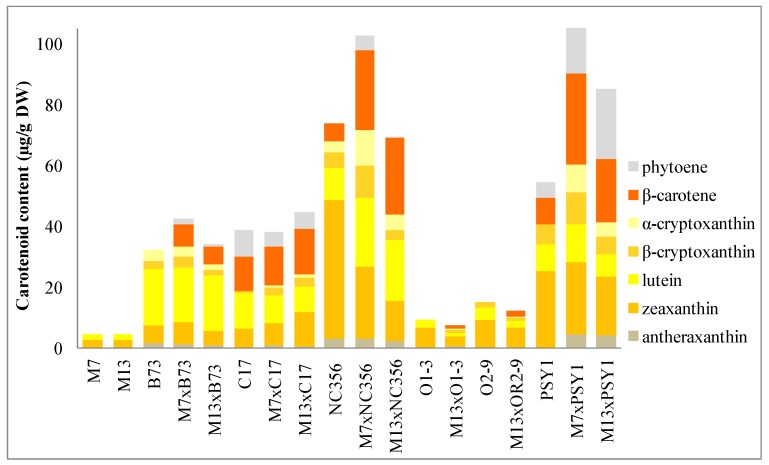
Carotenoid profiles in the endosperm (30 DAP) of parental lines M7, M13, B73, C17, NC356, O1-3, O2-9, PSY1, and the corresponding hybrids.

The endosperm carotenoid content and composition in transgenic lines M7 and M13 was similar to wild-type M37W plants. The transgenic lines accumulated ~4.5 µg/g dry weight (DW) total carotenoids compared to ~3 µg/g DW in the M37W parent. In both transgenic lines, zeaxanthin was much more abundant than lutein. Traces of antheraxanthin, a metabolic derivative of zeaxanthin (Figure 1), were detected in line M13 but not M7 (Figure 3, Table S1).

Among the selected inbred lines, B73 and C17 accumulated large quantities of β,ε-carotenoids [α-carotene (β,ε-carotene) and its derived xanthophylls] but low quantities of β,β-carotenoids [β-carotene (β,β-carotene) and its derived xanthophylls], and had similar total carotenoid levels in the endosperm but different carotenoid profiles. In line B73 (total carotenoid content ~32 µg/g DW), lutein was the predominant carotenoid (~19 µg/g DW) followed by zeaxanthin (~6 µg/g DW), α-cryptoxanthin (~4 µg/g DW), β-cryptoxanthin (~3 µg/g DW), and antheraxanthin (~2 µg/g DW). In contrast, line C17 (total carotenoid content ~39 µg/g DW) accumulated more lutein (~12 µg/g DW), β-carotene (~11 µg/g DW), and phytoene (~9 µg/g DW) than zeaxanthin (~6 µg/g DW), and also contained traces of β-cryptoxanthin (~1 µg/g DW).

The total carotenoid content of hybrids derived from lines B73 and C17 did not differ substantially from the levels detected in the inbred parents. In hybrid M7 × B73, the total carotenoid content increased by 1.3-fold to ~42 µg/g DW, whereas in hybrid M13 × B73 the total carotenoid content increased by 1.1-fold to ~34 µg/g DW. In hybrid M13 × C17, the total carotenoid content increased by 1.1-fold to ~45 µg/g DW, and the total carotenoid content of the M7 × C17 hybrid was ~38 µg/g DW, very slightly lower than the C17 parent. Although the total carotenoid levels showed little variation, we observed interesting changes in the carotenoid profiles of the hybrids compared to their parents. There were no significant changes in the level of β,ε-carotenoids in the B73 hybrids (M7 × B73 and M13 × B73), with lutein remaining the predominant carotenoid (18–19 µg/g DW) and lower levels of α-cryptoxanthin (2–3 µg/g DW). In contrast, the M7 × C17 and M13 × C17 hybrids accumulated traces of α-cryptoxanthin (~1 µg/g DW) that were not detected in the C17 parent, and lutein levels declined to 8–9 µg/g DW, representing a 1.3–1.4-fold decrease compared to C17.

The β,β-carotenoid profiles were particularly interesting because there were variations between different hybrids of the same parental line. In M7 × B73, the amount of zeaxanthin in the endosperm increased to 7 µg/g DW (1.2-fold increase compared to B73), but in M13 × B73 it fell to 5 µg/g DW (1.3-fold decrease compared to B73). The level of β-cryptoxanthin declined in both hybrids, to ~3 µg/g DW (1.2-fold decrease) and ~2 µg/g DW (1.4-fold decrease), respectively. Similarly, the level of antheraxanthin declined in both hybrids, to 1 µg/g DW (1.3-fold decrease) and 1 µg/g DW (1.7-fold decrease), respectively. Finally, although β-carotene was not detected in the B73 parent, the hybrids accumulated relatively large amounts (~6 µg/g DW) as well as traces of phytoene (~1 µg/g DW). However, in the C17 hybrids, β-carotene was the predominant carotenoid, increasing to ~13 µg/g DW (1.1-fold increase compared to C17) and to ~15 µg/g DW (1.2-fold increase) in the M7 × C17 and M13 × C17 hybrids, respectively. Furthermore, zeaxanthin levels increased to ~7 µg/g DW (1.2-fold increase) and 11 µg/g DW (~1.8-fold increase), respectively, and β-cryptoxanthin levels increased to ~2.6 µg/g DW (4.3-fold increase) and 2.8 µg/g (4.7-fold increase), respectively. The level of phytoene declined to ~5 µg/g DW (1.8-fold decrease) in both hybrids. No antheraxanthin was detected in the C17 parent, but 0.5–1 µg/g DW accumulated in the hybrids (Figure 3, Table S1).

The parental lines NC356, O1-3, O2-9 and PSY1 accumulated high levels of β,β-carotenoids but low levels of β,ε-carotenoids, and the total carotenoid levels and the profiles of different carotenoids differed considerably. NC356 accumulated the highest quantity of total carotenoids among the parental lines (~74 µg/g DW), predominantly zeaxanthin (~46 µg/g DW) and lutein (~11 µg/g DW), followed by β-carotene (~6 µg/g DW; 9% of total carotenoids), β-cryptoxanthin (~5 µg/g DW; 7% of total carotenoids), α-cryptoxanthin (~4 µg/g DW, 5% of total carotenoids), and antheraxanthin (~3 µg/g DW). O1-3, O2-9, and PSY1 are transgenic lines with the same M37W background. The carotenoid profiles of O1-3 and O2-9 (overexpressing the *A. thaliana ORANGE* gene, *AtOR*) were identical, but the total carotenoid content, and thus the abundance of individual carotenoids, was lower in line O1-3. We confirmed that the predominant carotenoid in line O1-3 (total carotenoid content ~11 µg/g DW) was zeaxanthin (~6 µg/g DW), followed by lutein (~3 µg/g DW), β-cryptoxanthin (~1 µg/g DW) and traces of antheraxanthin. The equivalent values for line O2-9 (total carotenoid content ~15 µg/g DW) were ~9 µg/g DW zeaxanthin, ~4 µg/g DW lutein, and ~2 µg/g DW β-cryptoxanthin. The total carotenoid content of line PSY1 was 54 µg/g DW, comprising ~25 µg/g DW zeaxanthin, ~9 µg/g DW β-carotene, ~9 µg/g DW lutein, ~7 µg/g DW β-cryptoxanthin, and ~5 µg/g DW phytoene. 

Hybrids derived from lines NC356 and PSY1 accumulated higher levels of carotenoids when crossed with line M7 than M13. The crosses M7 × O1-3 and M7 × O2-9 were not possible due to adverse field conditions so only the M13 × O1-3 and M13 × O2-9 hybrids were analyzed. The total carotenoid content of hybrid M7 × NC356 increased by 1.4-fold compared to NC356 (~103 µg/g DW), and that of M7 × PSY1 increased by 2-fold compared to PSY1 (~106 µg/g DW). The total carotenoid content of hybrid M13 × NC356 was similar to NC356 (~68 µg/g DW), but that of M13 × PSY1 increased by 1.6-fold compared to PSY1 (~85 µg/g DW). The total carotenoid content of hybrid M13 × O1-3 decreased by 1.4-fold compared to O1-3 (~8 µg/g DW) and that of M13 × O2-9 decreased by 1.2-fold compared to O2-9 (~12 µg/g DW).

The level of α-cryptoxanthin increased in all six hybrids (M7 × NC356, M13 × NC356, M13 × O1-3, M13 × O2-9, M7 × PSY1, and M13 × PSY1) compared to the parental lines. M7 × NC356 and M13 × NC356 accumulated ~12 and ~5 µg/g DW, representing 3.2-fold and 1.4-fold increases, respectively, compared to NC356. Although M13 × O1-3 and M13 × O2-9 accumulated only traces of α-cryptoxanthin, the parents O1-3 and O2-9 were entirely devoid of this carotenoid. More importantly, PSY1 also contained no traces of α-cryptoxanthin, but the hybrids M7 × PSY1 and M13 × PSY1 accumulated ~9 and ~5 µg/g DW, respectively. 

The hybrids showed diverse lutein profiles. In M7 × NC356, the lutein level increased to ~23 µg/g DW (2.1-fold more than NC356), and in M13 × NC356 it increased to ~20 µg/g DW (1.9-fold more than NC356). In contrast, the lutein level in M13 × O1-3 decreased to ~1 µg/g DW (2.4-fold less than O1-3), and in M13 × O2-9 it decreased to ~2 µg/g DW (1.8-fold less than O2-9). Interestingly, the lutein content of M7 × PSY1 increased to ~12 µg/g DW (1.4-fold more than PSY1), but in M13 × PSY1 it decreased to ~7 µg/g DW (1.2-fold less than PSY1). 

The β-cryptoxanthin content of the M7 hybrids was always higher than the corresponding parents, whereas in the M13 hybrids it was always lower. In both M7 × NC356 and M7 × PSY1, the level of β-cryptoxanthin was ~11 µg/g DW (2.1-fold more than NC356, and 1.6-fold more than PSY1). In M13 × NC356, M13 × O1-3, M13 × O2-9, and M13 × PSY1, β-cryptoxanthin accumulated to ~3 µg/g DW (1.7-fold less than NC356), ~1 µg/g DW (1.4-fold less than O1-3), ~1 µg/g DW (1.6-fold less than O2-9), and ~6 µg/g DW (1.1-fold less than PSY1), respectively. 

Zeaxanthin levels decreased in most of the hybrids compared to their parents, with the exception of M7 × PSY1. M7 × NC356 and M13 × NC356 accumulated ~24 and ~13 µg/g DW zeaxanthin (1.9-fold and 3.4-fold less than NC356), respectively. M13 × O1-3 and M13 × O2-9 accumulated ~3 and ~7 µg/g DW zeaxanthin, respectively (1.9-fold less than O1-3 and 1.4-fold less than O2-9). Finally, M7 × PSY1 accumulated ~24 µg/g DW zeaxanthin, which was similar to the PSY1 parent line, and M13 × PSY1 accumulated ~19 µg/g DW zeaxanthin (1.3-fold less than PSY1).

The β-carotene content showed remarkable increases in all six hybrids compared to the corresponding parents. M7 × NC356 accumulated ~26 µg/g DW (4.4-fold more than NC356), M13 × NC356 accumulated ~25 µg/g DW (4.3-fold more than NC356), M7 × PSY1 accumulated ~30 µg/g DW (3.4-fold more than PSY1), and M13 × PSY1 accumulated ~21 µg/g DW (2.4-fold more than PSY1). M13 × O1-3 and M13 × O2-9 accumulated ~1 and ~2 µg/g DW β-carotene, respectively, even though no traces of this carotenoid were found in the parents.

The upstream carotenoid phytoene, which was not detected in NC356, accumulated to ~5 µg/g DW in M7 × NC356 and trace amounts were also found in M13 × NC356. In M7 × PSY1 and M13 × PSY1, the phytoene levels increased to ~16 and ~23 µg/g DW (3.1-fold and 4.6-fold more than PSY1), respectively. Antheraxanthin, which was not detected in O2-9 or PSY1, accumulated to trace amounts in M13 × O2-9 and to ~5 µg and 4 µg/g DW in M7 × PSY1 and M13 × PSY1, respectively. The antheraxanthin content of the hybrids M7 × NC356 and M13 × O13 was similar to their parents, but the antheraxanthin content of the M13 × NC356 hybrid was ~2 µg/g DW, which was 1.4-fold less than NC356 (Figure 3, Table S1).

### 2.3. Analysis of Transgene and Endogenous Gene Expression in Hybrids Derived from BCH-Silenced Parents

**Figure 4:**
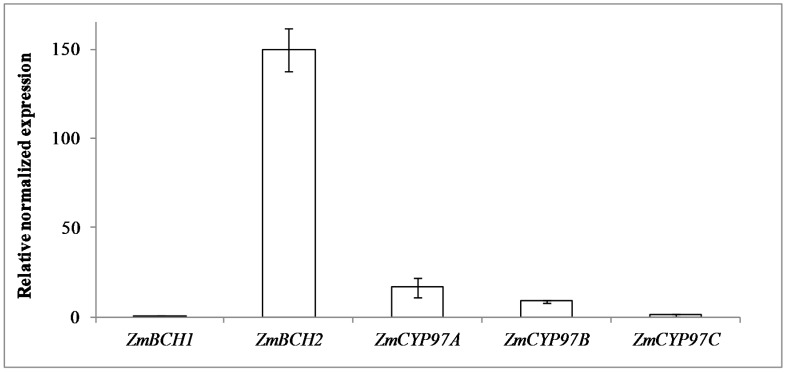
Expression of the endogenous hydroxylase genes *ZmBCH1*, *ZmBCH2*, *ZmCYP97A*, *ZmCYP97B*, and *ZmCYP97C* in wild-type (M37W) maize endosperm (30 DAP), normalized against *actin* mRNA, relative to *ZmBCH1* and presented as the mean of three replicates ± SE. Abbreviations: *ZmBCH1*, *carotenoid β-hydroxylase 1*; *ZmBCH2*, *carotenoid β-hydroxylase 2*; *ZmCYP97A/B*, *carotenoid β-hydroxylase*; *ZmCYP97C, carotenoid ε-hydroxylase*.

Transgene and endogenous gene expression was investigated by real-time RT-PCR and mRNA blot. *AtOR* expression was confirmed in the parental lines O1-3 and O2-9, and the corresponding hybrids M13 × O1-3 and M13 × O2-9. *ZmPSY1* expression was confirmed in the PSY1 line and the corresponding hybrids M7 × PSY1 and M13 × PSY1 (Figure S1). A comparison of endogenous *ZmBCH1*, *ZmBCH2*, *ZmCYP97A*, *ZmCYP97B*, and *ZmCYP97C* transcript levels relative to *ZmBCH1* revealed that the *ZmBCH1* and *ZmCYP97C* transcripts accumulated to similar levels in M37W endosperm. The levels of *ZmCYP97A* and *ZmCYP97B* expression in wild-type plants were 18-fold and 9-fold higher, respectively, than *ZmBCH1* expression, and *ZmBCH2* mRNA was 150-fold more abundant than *ZmBCH1* mRNA (Figure 4).

**Figure 5:**
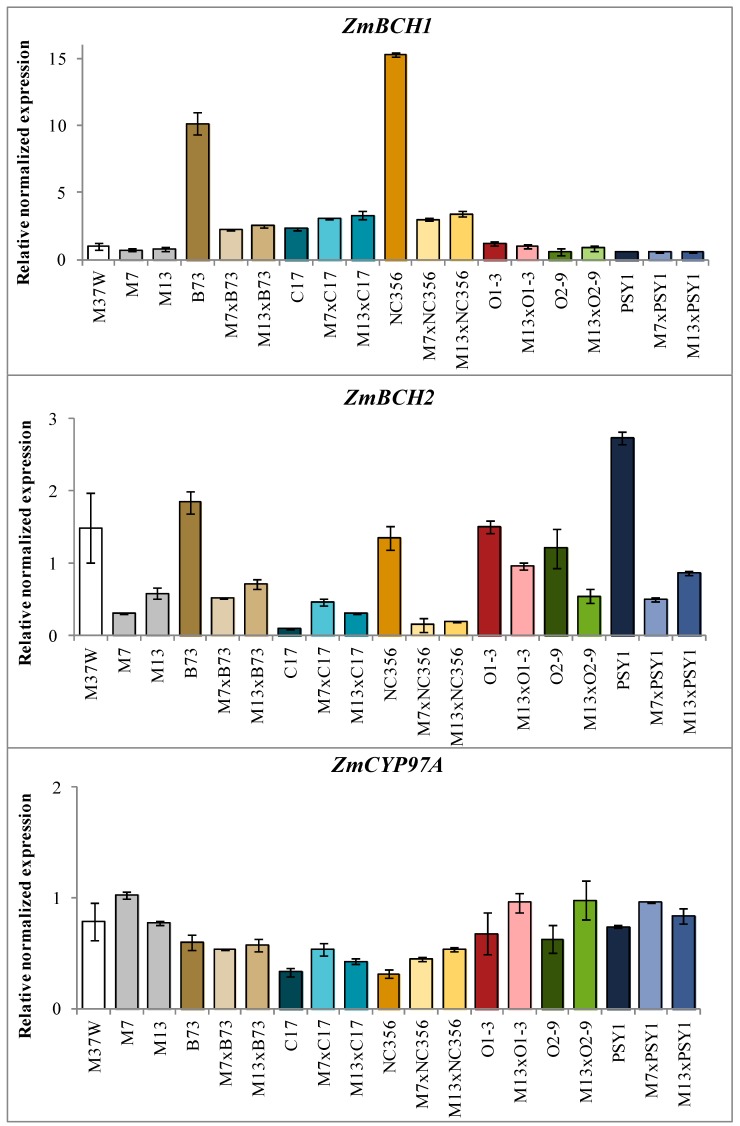

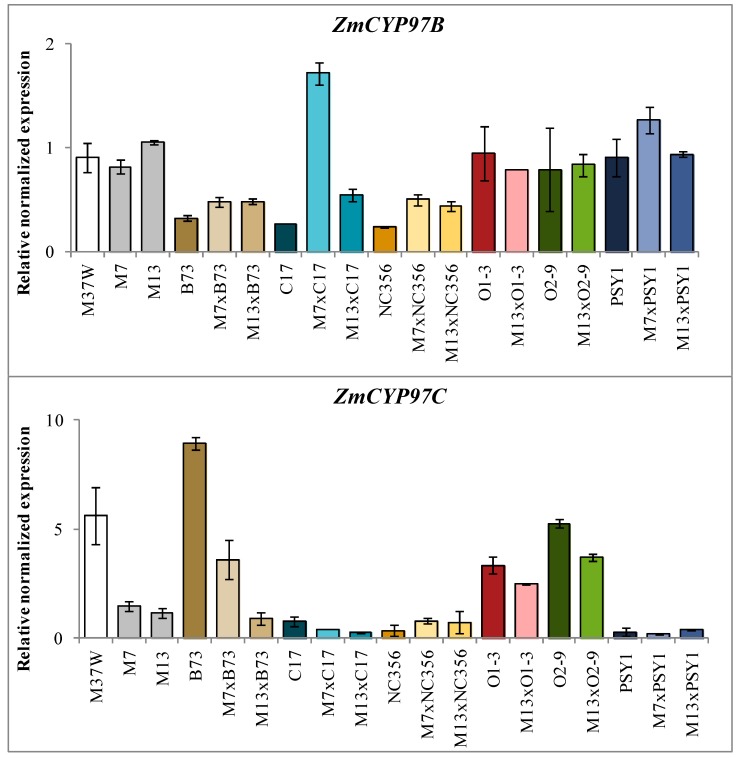
Expression of the endogenous hydroxylase genes *ZmBCH1*, *ZmBCH2*, *ZmCYP97A*, *ZmCYP97B,* and *ZmCYP97C* in 30 DAP maize endosperm, normalized against actin and relative to the expression levels in wild-type M37W maize and presented as the mean of three replicates ± SE. Abbreviations: *ZmBCH1*, *carotenoid β-hydroxylase 1*; *ZmBCH2*, *carotenoid β-hydroxylase 2*; *ZmCYP97A/B*, *carotenoid β-hydroxylase*; *ZmCYP97C, carotenoid ε-hydroxylase*.

The expression of the endogenous hydroxylase genes *ZmBCH1*, *ZmBCH2*, *ZmCYP97A*, *ZmCYP97B,* and *ZmCYP97C* was analyzed at 30 days after pollination (DAP) in the endosperm of the M37W, B73, C17, NC356, O1-3, O2-9 and PSY1 lines, and the corresponding hybrids generated with M7 and M13 (Figure 5). *ZmBCH1* and *ZmBCH2* expression was downregulated in both transgenic lines (~2-fold in M7 and ~1.5-fold in M13) compared to wild-type plants. The levels of *ZmCYP97A* and *ZmCYP97B* mRNA were similar in M7, M13, and M37W, but *ZmCYP97C* levels were ~3.5-fold lower in the transgenic lines compared to M37W (Figure 5). 

*ZmBCH1* was expressed at the highest levels in parental lines B73 and NC356 (~10-fold and ~15-fold higher, respectively, than in the RNAi lines). In the hybrids derived from these parents (M7 × B73, M13 × B73, M7 × NC356, and M13 × NC356), *ZmBCH1* expression was partially silenced (~5-fold lower than B73 and NC356). In line C17, *ZmBCH1* mRNA was only 3-fold more abundant than in lines M7 and M13, and the corresponding hybrids M7 × C17 and M13 × C17 produced 1.3-fold more *ZmBCH1* mRNA than the C17 parent. Endogenous *ZmBCH1* expression was similar in lines O1-3, O2-9, PSY1, and the original wild-type progenitor M37W, but *ZmBCH1* expression was not suppressed in the corresponding hybrids (M13 × O1-3, M13 × O2-9, M7 × PSY1, and M13 × PSY1) (Figure 5).

*ZmBCH2* was expressed at the highest levels in parental lines B73, NC356, and PSY1 (approximately 2-fold, 1.6-fold, and 3.5-fold higher levels, respectively, compared to M13). In the M13 hybrids, *ZmBCH2* was strongly downregulated compared to the levels in the wild-type parents (approximately 3-fold, 7-fold, and 3.5-fold compared to B73, NC356, and PSY1, respectively). *ZmBCH2* expression in M13 × O1-3 and M13 × O2-9 was also reduced (~2-fold) compared to parental lines O1-3 and O2-9. Because *ZmBCH2* levels in M7 were lower than in M13, hybrids derived from M7 line were higher downregulated than hybrids derived from M13. In contrast, *ZmBCH2* expression in parental line C17 was ~4-fold lower compared to lines M7 and M13, and the corresponding hybrids M7 × C17 and M13 × C17 accumulated similar levels of *ZmBCH2* mRNA compared to M7 and M13 parents (Figure 5).

*ZmCYP97C* transcript levels were ~9-fold higher in parental line B73 compared to M7 and M13, but in the corresponding hybrids (M7 × B73 and M13 × B73) they were ~2.5-fold and ~9-fold lower than B73, respectively (Figure 5). Similarly, *ZmCYP97C* levels were ~4.5-fold and ~6-fold higher in O1-3 and O2-9, respectively, compared to the M7 and M13 parents, and the corresponding hybrids (M13 × O1-3 and M13 × O2-9) accumulated ~1.3-fold lower levels of *ZmCYP97C* mRNA than the O1-3 and O2-9 parents. *ZmCYP97C* mRNA levels in line C17 were similar to M7 and M13, but in the M7 × C17 and M13 × C17 hybrids they were 2-fold lower than in C17. However, *ZmCYP97C* mRNA levels in line NC356 were 4-fold lower than in M7 and M13, but the corresponding hybrids (M7 × NC356 and M13 × NC356) accumulated 2-fold higher levels of *ZmCYP97C* mRNA than NC356. *ZmCYP97C* mRNA levels in line PSY1 were 3-fold lower than in the M7 and M13 parents, and remained at this level in the hybrids M7 × PSY1 and M13 × PSY1 (Figure 5). The levels of *ZmCYP97A* and *ZmCYP97B* mRNA showed little variation between the parental lines and the corresponding hybrids (Figure 5).

## 3. Discussion

### 3.1. Silencing the Endogenous ZmBCH1 and ZmBCH2 Genes Causes β-Carotene to Accumulate in the Endosperm of Diverse Maize Hybrids

Transgenic maize plants in which both *ZmBCH1* and *ZmBCH2* were silenced simultaneously were generated because the RNAi cassette targeted a sequence conserved in both genes with 96.4% identity (Figure 2A,B). The potency of *ZmBCH2* silencing varied among the transgenic lines, and we selected one line (M7) with near-complete *ZmBCH2* silencing and another (M13) with partial *ZmBCH2* silencing. *ZmBCH1* expression was already much lower than *ZmBCH2* in the wild-type M37W endosperm and it declined by 2-fold in both M7 and M13. The M37W inbred line used for transformation only accumulates traces of carotenoids because the *ZmPSY1* gene is expressed at low levels in the endosperm [28,29]. Consequently, primary transformants in an M37W genetic background cannot be used to evaluate the impact of *BCH* gene silencing in the endosperm. We therefore introgressed the RNAi cassette present in lines M7 and M13 into different maize backgrounds selected on the basis of their endosperm carotenoid content and composition, to determine how the near-complete or partial silencing of *ZmBCH2* affected carotenoid accumulation.

The carotenoid profiles of hybrids generated using transgenic lines M7 and M13 revealed diverse carotenoid profiles (Figure 3, Table S1). Silencing *ZmBCH1* and *ZmBCH2* had a more profound impact on the β,β-carotenoid branch, given that the endosperm of parental lines B73, O1-3, and O2-9 contains no β-carotene but the corresponding hybrids accumulated up to ~5 µg/g DW of this molecule. Furthermore, the NC356 and PSY1 hybrids accumulated up to 3.4-fold more β-carotene than their corresponding parents. However, no significant changes in β-carotene levels were detected in the C17 hybrids because *ZmBCH2* is already expressed at minimal levels in the C17 parent (Figure 3). The levels of β-cryptoxanthin increased in all C17 hybrids but decreased in all O1-3 and O2-9 hybrids. Interestingly, the levels of β-cryptoxanthin increased when B73, NC356 and PSY1 were hybridized with the strongly-silenced line M7 but decreased when the same parents were hybridized with the partially silenced line M13, suggesting that the degree of BCH activity is a key determinant of β-cryptoxanthin levels. The M7 parent has a lower hydroxylation capacity than M13, so the monohydroxylated carotenoid β-cryptoxanthin accumulates at the expense of the dihydroxylated carotenoid zeaxanthin. Accordingly, zeaxanthin levels decreased when NC356, O1-3, and O2-9 were hybridized with either of the RNAi lines, but increased in all hybrids of B73 and PSY1, showing a correlation between the depleted zeaxanthin pool and the increase in β-carotene levels due to *ZmBCH2* downregulation. This agrees with previous reports that BCH2 is the key determinant of the conversion of β-carotene into zeaxanthin in maize endosperm based on QTL mapping, GWAS and functional analysis [16,17,18,21,22].

In the α-branch of the pathway, the β-hydroxylation of α-cryptoxanthin produces lutein. We found that α-cryptoxanthin accumulated in hybrids of C17, O1-3, O2-9, and PSY1 even though the parental lines do not produce this metabolite. In the M7 × B73 hybrid, the level of α-cryptoxanthin was similar to that detected in the B73 parent, but the M13 × B73 hybrid produced more α-cryptoxanthin than B73. Furthermore, both NC356 hybrids accumulated more α-cryptoxanthin than the NC356 parent. The B73 and NC356 lines accumulated the highest levels of *ZmBCH1* mRNA, but the presence of α-cryptoxanthin suggests that additional hydroxylase activity is required for complete conversion to lutein. The quantity of lutein was reduced in the hybrids of lines C17, O1-3, and O2-9 compared to the parent lines, but increased in the NC356 hybrids and did not change in the PSY1 hybrids, again suggesting that additional β-hydroxylase activity (most likely provided by ZmCYP97A and/or ZmCYP97B) is necessary for the complete conversion of α-cryptoxanthin into lutein. ZmCYP97A and/or ZmCYP97B orthologs in *A. thaliana* [9,10], tomato [13], and rice [14,15] are known to be involved in lutein biosynthesis.

### 3.2. ZmBCH1 and ZmBCH2 Silencing Affects the Expression of CYP-Type ε-Hydroxylase Gene

In the M37W endosperm, *ZmBCH2* is expressed more strongly than *ZmBCH1*, *ZmCYP97A*, *ZmCYP97B*, and *ZmCYP97C* (Figure 4). *ZmBCH1* is more important for the β-ring hydroxylation of α-carotene than β-carotene, because polymorphisms in this gene correlated more with variations in α-carotene levels rather than β-carotene levels [19]. *ZmBCH1* mRNA levels varied widely in the different parental lines, with some lines accumulating up to 15-fold higher levels of this transcript than M37W. *ZmBCH1* mRNA levels were up to 5-fold lower in the B73 and NC356 hybrids than their parents, suggesting that *ZmBCH1* was strongly suppressed by the introgressed RNAi construct. However, there was little impact on *ZmBCH1* mRNA levels in hybrids of O1-3, O2-9, and PSY1, and *ZmBCH1* mRNA levels in C17 hybrids were higher than in the C17 and M37W parents, suggesting that endogenous *ZmBCH1* alleles might influence the effectiveness of the RNAi construct introgressed into these hybrids. The strong suppression of *ZmBCH1* expression in hybrids of B73 and NC356 compared to the corresponding parental lines did not correlate with higher α-cryptoxanthin and lower lutein levels, which suggests that additional carotenoid β-hydroxylase activity (probably *ZmCYP97A* and/or *ZmCYP97B*, as stated above) may be required for the complete conversion of α-cryptoxanthin into lutein. *ZmBCH2* mRNA levels varied by only 2.5-fold among all the parental lines and hybrids. However, the *ZmBCH2* locus has a strong impact on β-carotene accumulation in the endosperm [16,18] because it is the only hydroxylase gene strongly expressed in this tissue [17]. In most hybrids, *ZmBCH2* mRNA levels were lower than in the corresponding parental lines, but the C17 hybrids were exceptional: the level of *ZmBCH2* mRNA was lower than in the RNAi lines but higher than the C17 parent.

The different degrees of gene silencing in M7 and M13 also manifested in the hybrids derived from these lines. The levels of *ZmCYP97A* and *ZmCYP97B* mRNA in the parent lines and hybrids were too low to detect obvious differences, and any differences could reflect natural variations rather than the consequences of *BCH* gene silencing. This low level of variability also suggests that *ZmCYP97A* and *ZmCYP97B* are tightly regulated. The levels of *ZmCYP97A* and *ZmCYP97B* were similar in hybrids and their parents, whereas the carotenoid ε-hydroxylase gene *ZmCYP97C* varied by up to 19-fold, with lines B73 and C17 representing the extremes. *ZmCYP97C* was downregulated in the hybrids of B73, C17, O1-3, and O2-9 compared to the parental lines, but not in hybrids of NC356 or PSY1. Interestingly, the NC356 or PSY1 hybrids did not show a reduction in lutein levels, suggesting that the downregulation of *BCH* gene expression may have a feedback effect on the genes encoding carotenoid ε-hydroxylases.

## 4. Materials and Methods

### 4.1. Gene Cloning and Vector Construction

The *ZmBCH2* cDNA fragment was cloned from maize (*Zea mays* L. cv. B73) endosperm total RNA by RT-PCR using forward primer 5′-GGA ATT CTC TAG ACT ATC GCT TCA GCT GGC AAA TGG AG-3′ (EcoRI and XbaI sites are underlined) and reverse primer 5′-GAC TAG TGG ATC CAA CTT GTC CAT GTG GTG TAT CTT G-3′ (BamHI and SpeI sites are underlined) based on sequence information in GenBank (accession number: AY844958). The *ZmBCH2* sequence was transferred to the pHorP vector containing the barley D-hordein promoter, a 300-bp *gus*A gene fragment, and the ADP-glucose pyrophosphorylase terminator (ADGPP) using restriction enzymes XbaI and BamHI to yield the intermediate vector pHorP-ZmBCH2 sense. In a second step, the intermediate vector was digested with SpeI and EcoRI to introduce the antisense *ZmBCH2* fragment between the *gus*A gene and ADGPP terminator, resulting in pHorP-RNAi-ZmBCH2 (Figure S2).

### 4.2. Maize Transformation and Plant Growth

We transformed 14-day-old immature zygotic embryos of South African elite white maize inbred M37W by bombarding them with gold particles coated with the pHorP-RNAi-ZmBCH2 construct and the *bar* gene for selection. Transgenic maize lines M1, M7, M9, and M13 were obtained following the procedure described by Zhu et al. (2008) [28]. Transgenic maize lines expressing *AtOR* (O1-3 and O2-9) and *ZmPSY1* (PSY1) were described in our earlier studies [30]. Three inbred lines selected on the basis of their carotenoid profiles were obtained from the USDA or CSIC (Table S2). Homozygous M7, M13, O1-3, O2-9, B73, C17, and NC356 plants were self-pollinated as controls, or out-crossed with lines M7 and M13 (pollen donor) to obtain the hybrids M7 × B73, M7 × C17, M7 × NC356, M7 × PSY1, M13 × B73, M13 × C17, M13 × NC356, M13 × O1-3, M13 × O2-9, and M13 × PSY1. For further analysis, endosperm samples were taken from immature seeds 30 DAP, frozen in liquid nitrogen, and stored at −80 °C.

### 4.3. RNA Extraction and Expression Analysis

Total RNA was extracted for mRNA blot analysis and cDNA synthesis as previously described [29]. Quantitative real-time PCR (qRT-PCR) was carried out in triplicate using the primers listed in Table S3. Real-time RT-PCR was performed on a BioRad CFX96TM system using 25-µL mixtures containing 10 ng of synthesized cDNA, 1× iQ SYBR Green Supermix (BioRad, Hercules, CA, USA), and 0.2 µM forward and reverse primers. Relative expression levels were calculated on the basis of serial dilutions of cDNA (125–0.2 ng), which were used to generate standard curves for each gene. Cycling conditions consisted of a single incubation step at 95 °C for 5 min followed by 30 cycles of 95 °C for 10 s, 58 °C for 35 s, and 72 °C for 15 s. Specificity was confirmed by product melt curve analysis over the temperature range 50–90 °C with fluorescence acquired after every 0.5 °C increase, and the fluorescence threshold value and gene expression data were calculated with BioRad CFX manager software version 3.1. Values represent the mean of three biological replicates ± SE. Amplification efficiencies were compared by plotting the Δ*C*_t_ values of different primer combinations of serial dilutions against the log of starting template concentrations using the CFX96TM software. Differences among samples were observed by mean ± SE.

### 4.4. Carotenoid Extraction and UPLC (Ultra Performance Liquid Chromatography) Analysis

Carotenoids were extracted from 30 DAP maize endosperm for UPLC analysis in triplicate as described by Berman et al. [30]. Data were calculated from three replicates with error bars representing the standard error. Differences among samples were observed by mean ± SE.

## 5. Conclusions

The RNAi-ZmBCH2 cassette in transgenic lines M7 and M13 was introgressed into several maize genetic backgrounds with different carotenoid profiles. Knocking down endogenous *ZmBCH2* expression by RNAi increased β-carotene levels in hybrids of the parental lines B73, N356, O1-3, O2-9, and PSY1, regardless of whether or not the endogenous *ZmBCH1* gene was also affected. However, we detected no change in the level of β-carotene in hybrids of C17 (M7 × C17 and M13 × C17) as compared to the C17 parent because *ZmBCH2* transcript levels were already very low. These results indicate that *ZmBCH2* is the critical enzyme responsible for the conversion of β-carotene to zeaxanthin via β-cryptoxanthin in maize endosperm, and that its activity may be intimately linked with that of the carotenoid ε-hydroxylase encoded by *ZmCYP97C*.

## Supplementary Materials

Supplementary materials can be found at www.mdpi.com/1422-0067/18/12/2515/s1.
