# Supplementary material for: The Silencing of Carotenoid β-Hydroxylases by RNA Interference in Different Maize Genetic Backgrounds Increases the β-Carotene Content of the Endosperm
Source: Int J Mol Sci. 2017 Nov 24;18(12):2515. doi: 10.3390/ijms18122515 (PMC5751118; doi:10.3390/ijms18122515)
Supplement: Supplementary file 1 [file ijms-18-02515-s001.pdf]

International Journal of *Molecular Sciences*

MDPI

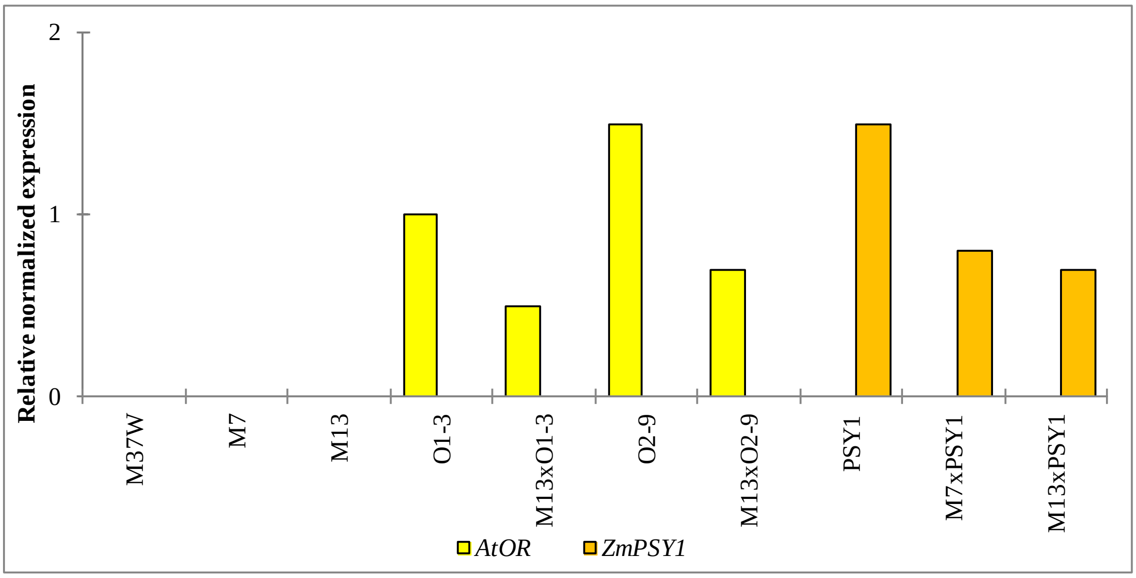

Figure S1. Transgene expression normalized against actin in wild-type (M37W) and transgenic lines presented as means of three replicates plus standard errors.

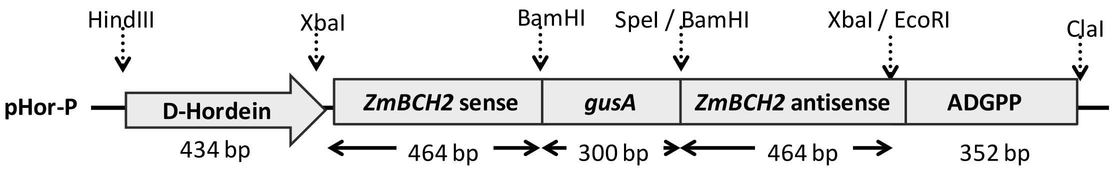

Figure S2 Schematic representation of pHorP-RNAi-ZmBCH2.

Table S1. Carotenoid content and composition of wild-type (M37W), M7, M13, B73, C17, NC356, O1-3, O2-9 and PSY1 parents and the corresponding hybrids at 30 DAP (μg/g DW±SE). The percentage of individual carotenoids in the endosperm is shown in brackets. Abbreviations: anthera, antheraxanthin; zea, zeaxanthin; lut, lutein; β-crypto, β-cryptoxanthin; α-crypto, α-cryptoxanthin; β-caro, β-carotene; phyto, phytoene; total caro, total carotenoids.

Plant line	anthera μg/g DW (%)	zea μg/g DW (%)	lut μg/g DW (%)	β-crypto μg/g DW (%)	α-crypto μg/g DW (%)	β-caro μg/g DW (%)	phyto μg/g DW (%)	Total caro μg/g DW
WT	0.0±0.00 (0)	2.0±0.10 (59)	1.1±0.00 (32)	0.0±0.00 (0)	0.0±0.00 (0)	0.0±0.00 (0)	0.3±0.00 (9)	3.4
M7	0.0±0.00 (0)	2.8±0.27 (60)	1.9±0.19 (40)	0.0±0.00 (0)	0.0±0.00 (0)	0.0±0.00 (0)	0.0±0.00 (0)	4.7
M13	0.3±0.02 (6)	2.5±0.19 (57)	1.7±0.14 (37)	0.0±0.0 (0)	0.0±0.00 (0)	0.0±0.00 (0)	0.0±0.00 (0)	4.5
B73	1.5±0.09 (5)	6.1±0.38 (19)	18.6±0.93 (57)	2.8±0.23 (8)	3.6±0.18 (11)	0.0±0.00 (0)	0.0±0.00 (0)	32.6
M7x B73	1.2±0.04 (3)	7.4±0.23 (17)	18.1±0.18 (43)	3.3±0.24 (8)	3.5±0.20 (8)	7.1±0.45 (17)	1.8±0.12 (4)	42.4
M13x B73	0.9±0.05 (3)	4.6±0.06 (13)	18.5±0.04 (54)	1.8±0.24 (5)	1.8±0.05 (5)	5.8±0.21 (17)	0.7±0.02 (2)	34.1
C17	0.0±0.00	6.2±0.20	12.0±0.30	0.6±0.03	0.0±0.00	11.4±0.90	8.7±0.02	38.9

	(0)	(16)	(31)	(1)	(0)	(29)	(22)	
M7x C17	0.8±0.01 (2)	7.3±0.20 (19)	9.0±0.21 (24)	2.6±0.15 (7)	1.0±0.01 (3)	12.6±0.17 (33)	4.8±0.13 (13)	38.1
M13x C17	0.4±0.01 (1)	11.2±0.22 (25)	8.5±0.18 (19)	2.8±0.03 (6)	1.2±0.01 (3)	14.9±0.51 (34)	5.4±0.11 (12)	44.4
NC356	3.0±0.19 (4)	45.5±0.78 (62)	10.8±0.17 (15)	5.0±0.25 (7)	3.7±0.16 (5)	5.9±0.41 (8)	0.0±0.00 (0)	73.9
M7x NC356	3.0±0.04 (3)	23.7±0.04 (23)	22.7±0.5 (22)	10.5±0.19 (10)	11.9±0.28 (12)	26.0±1.00 (25)	4.9±0.15 (5)	102.7
M13x NC356	2.2±0.00 (3)	13.2±0.17 (19)	20.2±0.10 (29)	3.0±0.19 (4)	5.2±0.16 (7)	25.3±1.40 (36)	0.4±0.00 (1)	69.5
O1-3	0.2±0.02 (2)	6.5±0.38 (61)	2.6±0.08 (25)	1.3±0.01 (12)	0.0±0.00 (0)	0.0±0.00 (0)	0.0±0.00 (0)	10.6
M13x O1-3	0.5±0.03 (7)	3.4±0.36 (45)	1.1±0.1 (14)	0.9±0.05 (12)	0.5±0.02 (6)	1.2±0.03 (15)	0.0±0.00 (0)	7.6
O2-9	0.0±0.00 (0)	9.3±2.02 (62)	3.9±0.35 (26)	1.9±0.40 (13)	0.0±0.00 (0)	0.0±0.00 (0)	0.0±0.00 (0)	15.1
M13x OR2-9	0.8±0.06 (7)	6.5±0.04 (53)	2.2±0.08 (18)	1.2±0.01 (9)	0.6±0.05 (5)	1.8±0.04 (15)	0.0±0.00 (0)	13.1
PSY1	0.0±0.00 (0)	25.5±2.14 (47)	8.5±0.35 (16)	6.6±0.29 (12)	0.0±0.00 (0)	8.7±0.75 (16)	5.0±0.18 (9)	54.3
M7x PSY1	4.5±0.05 (4)	23.8±0.19 (22)	12.3±0.17 (12)	10.5±0.25 (10)	9.3±0.09 (9)	29.9±0.49 (28)	15.7±0.10 (15)	106.0
M13x PSY1	4.2±0.3 (5)	19.2±0.25 (23)	7.3±0.15 (9)	5.9±0.12 (7)	4.6±0.03 (5)	20.7±0.09 (25)	23.0±0.18 (27)	84.9

Table S2 Maize lines with specific carotenoid profiles used to evaluate the effect of *BCH* gene silencing by RNAi.

Line	Genotype	Source	Carotenoid profile	References
B73	Inbred	USDA	High lutein<br>Very low β/ε ratio	(Harjes et al., 2008) (Vallabhaneni and Wurtzel, 2009)
C17	Inbred	USDA	High β-carotene<br>High β/ε ratio	(Yan et al., 2010)
NC356	Inbred	USDA	High zeaxanthin<br>Very high β/ε ratio	(Yan et al., 2010)
PSY1	Transgenic *ZmPSY1*	Applied plant biotechnology, Universitat de Lleida, Spain	High zeaxanthin and β-carotene<br>High β/ε ratio	(Berman et al., 2017)
O1-3	Transgenic *AtOR*	Applied plant biotechnology, Universitat de Lleida, Spain	High zeaxanthin<br>Medium β/ε ratio	(Berman et al., 2017)
O2-9	Transgenic AtOR	Applied plant biotechnology, Universitat de Lleida, Spain	High zeaxanthin<br>Medium β/ε ratio	(Berman et al., 2017)

USDA: United States Department of Agriculture; CSIC: Consejo Superior de Investigaciones Científicas

References

1. Harjes CE, Rocheford TR, Bai L, Brutnell TP, Kandianis CB, Sowinski SG, Stapleton AE, Vallabhaneni R, Williams M, Wurtzel ET, Yan J, Buckler ES (2008) Natural genetic variation in lycopene epsilon cyclase tapped for maize biofortification. Science. 319:330–333.
2. Vallabhaneni R, Gallagher CE, Licciardello N, Cuttriss AJ, Quinlan RF, Wurtzel ET. (2009) Metabolite sorting of a germplasm collection reveals the *hydroxylase 3* locus as a new target for maize provitamin A biofortification. Plant Physiol. 151:1635–1645.
3. Yan J, Kandianis CB, Harjes CE, Bai L, Kim E, Yang X, Skinner DJ, Fu Z, Mitchell S, Li Q, Fernandez MG, Zaharieva M, Babu R, Fu Y, Palacios N, Li J, Dellapenna D, Brutnell T, Buckler ES, Warburton ML, Rocheford T (2010) Rare genetic variation at *Zea mays crtRB1* increases β-carotene in maize grain. Nat Genet. 42:322–327.
4. Berman J, Zorrilla-López U, Medina V, Farré G, Sandmann G, Capell T, Christou P, Zhu C (2017) The Arabidopsis *ORANGE* (*AtOR*) gene promotes carotenoid accumulation in transgenic corn hybrids derived from parental lines with limited carotenoid pools. Plant Cell Rep. 36:933–945.

Table S3. Primer sequences used for qRT-PCR.

Gene	Forward	Reverse
ZmACTIN	5′-CGATTGAGCATGGCATTGT-3′	5′-CCCACTAGCGTACAACGAA-3′
ZmBCH1	5′-CCACGACCAGAACCTCCAGA-3′	5′-CATGGCACCAGACATCTCCA-3′
ZmBCH2	5′-GCTTGTTAGCAGTCCGGT-3′	5′-GAAAGGAGGATGGCGATAGAT-3′
ZmCYP97A	5′-CTGGAGCGTCTGAAAGTCA-3′	5′-GGACCAAATCCAAACGAGAT-3′
ZmCYP97B	5′-CTGAGGAGAAGGACTTGA-3′	5′-TCCACTGGTCTGTCTGCGAT-3′
ZmCYP97C	5′-GTTGACATTGGATGTGATTGG-3′	5′-AACCAACCTTCCAGTATGGC-3′
ZmPSY1	5′-CATCTTCAAAGGGGTCGTCA-3′	5′-CAGGATCTGCCTGTACAACA-3′
AtOR	5′- TTCTCTATCACCGCCCAAAAC-3′	5′- GCCATAGCCATTCCTGTGC-3′
